# Cardiac Magnetic Resonance Left Ventricular Filling Pressure Is Associated with NT-proBNP in Patients with New Onset Heart Failure

**DOI:** 10.3390/medicina59111924

**Published:** 2023-10-30

**Authors:** Hosamadin Assadi, Gareth Matthews, Bradley Chambers, Ciaran Grafton-Clarke, Mubien Shabi, Sven Plein, Peter P Swoboda, Pankaj Garg

**Affiliations:** 1 Department of Cardiovascular and Metabolic Health, Norwich Medical School, University of East Anglia, Norwich NR4 7TJ, UK; 2 Department of Cardiology, Norfolk and Norwich University Hospitals NHS Foundation Trust, Norwich NR4 7UY, UK; 3 Division of Biomedical Imaging, Leeds Institute of Cardiovascular and Metabolic Medicine, University of Leeds, Leeds LS2 9JT, UKs.plein@leeds.ac.uk (S.P.);

**Keywords:** left ventricular end-diastolic pressure, MRI, CMR, diastole, heart failure

## Abstract

*Background and Objectives*: Cardiovascular magnetic resonance (CMR) is emerging as an important imaging tool for sub-phenotyping and estimating left ventricular (LV) filling pressure (LVFP). The N-terminal prohormone of B-type natriuretic peptide (NT-proBNP) is released from cardiac myocytes in response to mechanical load and wall stress. This study sought to investigate if CMR-derived LVFP is associated with the serum levels of NT-proBNP and, in addition, if it provides any incremental prognostic value in heart failure (HF). *Materials and Methods*: This study recruited 380 patients diagnosed with HF who underwent same-day CMR and clinical assessment between February 2018 and January 2020. CMR-derived LVFP was calculated, as previously, from long- and short-axis cines. During CMR assessment, serum NT-proBNP was measured. The pathological cut-offs were defined as follows: NT-proBNP ≥ 125 pg/mL and CMR LVFP > 15 mmHg. The incidence of HF hospitalisation was treated as a clinical outcome. *Results*: In total, 305 patients had NT-proBNP ≥ 125 pg/mL. Patients with raised NT-proBNP were older (54 ± 14 vs. 64 ± 11 years, *p <* 0.0001). Patients with raised NT-proBNP had higher LV volumes and mass. In addition, CMR LVFP was higher in patients with raised NT-proBNP (13.2 ± 2.6 vs. 15.4 ± 3.2 mmHg, *p* < 0.0001). The serum levels of NT-proBNP were associated with CMR-derived LVFP (R = 0.42, *p* < 0.0001). In logistic regression analysis, this association between NT-proBNP and CMR LVFP was independent of all other CMR variables, including LV ejection fraction, LV mass, and left atrial volume (coefficient = 2.02, *p* = 0.002). CMR LVFP demonstrated an independent association with the incidence of HF hospitalisation above NT-proBNP (hazard ratio 2.7, 95% confidence interval 1.2 to 6, *p* = 0.01). *Conclusions:* A CMR-modelled LVFP is independently associated with serum NT-proBNP levels. Importantly, it provides an incremental prognostic value over and above serum NT-proBNP levels.

## 1. Introduction

Heart failure (HF) is a growing global health concern with an estimated prevalence of over 37.7 million individuals worldwide [1]. The non-invasive estimation of left ventricular filling pressure (LVFP) is crucial in diagnosing and managing HF [2]. Echocardiography remains the mainstay for the non-invasive assessment of LVFP [3]. However, the accuracy of echocardiographic indices in estimating LVFP has recently been debated. While echocardiography has been shown to be feasible and accurate in identifying patients with elevated LVFP, it is only sometimes consistent with invasive measurements, which are considered the gold standard [4]. In addition, the skill and experience of the individual responsible for the image and data acquisition of the ultrasound are critical factors. This includes having the ability to manipulate the transducer to obtain optimal image quality and Doppler flow velocity signals, as well as having knowledge of ultrasound instrument settings such as the transducer frequency, use of harmonics, mechanical index, depth, gain, time-gain-compensation, dynamic range, filtering, velocity scale manipulations, and display of received signals [5]. Furthermore, Doppler imaging is directionally limited. The maximum Doppler shift is detected when the ultrasound beam is parallel to the flow. The maximum detected velocity is reduced by a function of the cosine of the angle away from parallel. All flow indices should be interrogated with multiple imaging planes, and this further introduces noise and variability [6]. Hence, echocardiographic measurements, including the E/e’ ratio, have a poor to moderate predictive value for the estimation of invasively acquired left ventricular end-diastolic pressure (LVEDP) and PCWP, as demonstrated in patients with heart failure with preserved ejection fraction (HFpEF) [7]. Importantly, in a more recent work, Pak et al. demonstrated that 60% of patients with elevated LVFP by echocardiography had normal LVFP by invasive assessment—again questioning its reliability for an accurate assessment of LVFP [8]. In this large study (*n* = 1967), the authors concluded that current echocardiography approaches are unreliable in detecting elevated LVFP.

More recently, cardiovascular magnetic resonance (CMR) has been used to estimate LVFP. CMR already plays a key clinical role in sub-phenotyping HF [9,10,11]. CMR is also recognised for its ability to provide an accurate and highly reproducible assessment of cardiac volumes, mass, and the ejection fraction of the left and right ventricles, as stated by the American College of Cardiology and the American Heart Association. CMR can estimate LVFP in patients with suspected HF, and the CMR-modelled LVFP has been shown to have prognostic power [12]. It has demonstrated superiority to transthoracic echocardiography in classifying patients as having normal or raised filling pressures (76% vs. 25%), and was associated with an increased risk of death [3,13]. In addition to its role in HF, CMR-derived LVFP can also be used to measure acute and dynamic changes in preloading conditions on the left ventricle during adenosine-administered first-pass perfusion CMR, where it has been observed to rise significantly [14].

The release of the N-terminal prohormone of B-type natriuretic peptide (NT-proBNP) stems from cardiac myocytes as a response to mechanical load and wall stress [15]. This secretion is predominantly initiated by myocardial stretching, a phenomenon closely linked to conditions like heart failure [16]. Studies have shown a strong correlation between NT-proBNP levels and LVFP, with higher levels of NT-proBNP associated with increased LVFP [17,18,19,20]. However, it remains unknown if CMR-derived LVFP is associated with NT-proBNP levels. Cardiovascular conditions such as atrial fibrillation and renal failure can cause elevated NT-proBNP levels, potentially confounding the interpretation of NT-proBNP measurements [21]. The European Society of Cardiology and the Heart Failure Association highlight these factors as significant impediments to the interpretation of NT-proBNP measurements in their 2016 guidelines [21]. Hence, there is a clinical need for more precise imaging biomarkers of LVFP that have added a clinical prognostic value over NT-proBNP levels. 

Thus, we carried out this study to (1) investigate whether CMR-derived LVFP is associated with the serum levels of NT-proBNP and (2) examine if the CMR-modelled LVFP has a better association with the incidence of HF than NT-proBNP levels.

## 2. Materials and Methods

### 2.1. Study Population

Between February 2018 and January 2020, adult patients seen in the cardiology clinic with a possible diagnosis of HF in the preceding 12 months, according to the European Society of Cardiology Heart Failure guidelines, were prospectively recruited.

The inclusion criteria established for participant recruitment in this study were as follows: (1) Adults: The study sought to enrol individuals who are 18 years of age or older. (2) Suspected Heart Failure: Individuals needed to present with suspected heart failure to be considered for inclusion in the study. (3) Ambulatory: The inclusion criterion of ambulatory status means that participants were required to have the ability to walk and move independently. In heart failure, this criterion typically implies that individuals could engage in regular daily activities without constant assistance or being bedridden. Ambulatory status is relevant because it helps ensure that participants have a certain level of physical functionality, which can be important in research studies involving heart failure management and assessment. This also meant that we were not able to recruit decompensated heart failure patients and only recruited patients in compensated physiological states into the study.

Participants were deemed ineligible for inclusion if they had a documented history of coronary artery disease, as defined by one or more of the following criteria: significant stenosis exceeding 70% observed during invasive angiography, confirmed prior myocardial infarction, previous percutaneous coronary intervention, or a history of coronary artery bypass grafting. Additionally, individuals presenting with symptoms consistent with angina pectoris, hypertrophic cardiomyopathy, or congenital heart disease were excluded. Furthermore, individuals with acute pathologies such as myocarditis, acute renal impairment, or any contraindications to CMR or gadolinium-based contrast agents were excluded from the study.

This study’s assessment of participant outcomes involved a comprehensive review of electronic hospital records. The primary endpoint of interest was the incidence of hospitalisation attributed to heart failure. This evaluation included a detailed examination of the participants’ medical histories and hospitalisation events to determine any instances where their clinical condition necessitated admission to a healthcare facility specifically for heart failure-related issues.

Reviewing the electronic hospital records entailed systematically analysing a wealth of medical data, including patient charts, diagnostic reports, treatment interventions, and discharge summaries. It aimed to identify any episodes where participants had experienced exacerbations of heart failure, leading to hospitalisation. Such hospitalisations could have been prompted by various factors, including acute exacerbations of heart failure symptoms, the need for specialised cardiac interventions, or complications arising from the management of heart failure.

Blood samples were collected concurrently with the intravenous cannulation procedure during the study, ensuring minimal disruption to the participants’ routines. These blood samples were immediately processed and then subjected to a carefully controlled freezing process, reaching a temperature of −70 °C. This sub-zero temperature was maintained to safeguard the stability and preservation of the serum until further analysis. The analysis of NT-proBNP was conducted from the defrosted serum samples using a Siemens Advia assay. All NT-proBNP measurements were carried out as part of a single batch analysis, ensuring consistency and uniformity in the laboratory procedures.

Written informed consent was obtained from all subjects involved in the study. The study protocol was approved by the National Research Ethics Service (17/YH/0300) in the United Kingdom in October 2017. The investigation aligns with the ethical principles delineated in the Declaration of Helsinki.

### 2.2. CMR Acquisition

All CMR studies were conducted using a 3 Tesla system (Siemens Magnetom Prisma, Erlangen, Germany). Participants were instructed to refrain from consuming caffeine for 24 h prior to their scheduled CMR examination. Each patient had an intravenous cannula inserted, and standard vital signs, including heart rate, blood pressure, and ECG, were continuously monitored throughout the procedure. The comprehensive CMR protocol, which lasted approximately 45 min, included baseline survey images, cine imaging in multiple planes, including an orthogonal long-axis and a stack of short-axis views, native and post-contrast T1 mapping, stress and rest perfusion, as well as late gadolinium enhancement (LGE) imaging. For the standard cines, we acquired 30 phases using a fast gradient echo sequence with 10–12 slices by either free-breathing with motion correction (MOCO) or 5-s breath-holds. For Native T1 mapping, we used a 5s3s modified Look-Locker inversion recovery (MOLLI) sequence involving three slices with an 11-s breath-hold. For stress perfusion imaging, intravenous adenosine was administered at 140 mcg/kg/min, during which blood pressure and ECG were monitored, and contrast (Gadovist^®^, Bayer Inc, Mississauga, ON, Canada) was injected if specific criteria were met. We used an identical sequence and geometry to the stress imaging for rest perfusion imaging, but without adenosine administration. Late gadolinium imaging was performed approximately 10 to 15 min after the final contrast injection administration with free breathing and motion correction. This imaging session maintained consistent long- and short-axis slice positions as in the cine imaging, employing a segmented inversion-recovery gradient echo sequence. Post-contrast T1 mapping was acquired following the contrast injection, utilising a 4s3s2s MOLLI sequence with a 12-s breath-hold. In cases of uncertainty regarding the presence of enhancement in the bright blood LGE images, a dark blood LGE sequence was acquired to provide additional clarity.

### 2.3. CMR Analysis

The assessment of cardiac volumes and the detection of late gadolinium enhancement were performed using the Circle CVI42 software (Circle CVI42 CMR analysis software version 5.17, Cardiovascular Imaging in Calgary, Calgary, AB, Canada). The process of manually outlining the endocardial and epicardial borders, with the exclusion of the papillary muscles, was conducted on the series of short-axis cine images. Left ventricular end-diastolic volume (LVEDV), left ventricular end-systolic volume (LVESV), right ventricular end-diastolic volume (RVEDV), and right ventricular end-systolic volume (RVESV) were subsequently calculated. Further computations were carried out to determine essential functional parameters, such as the left ventricular stroke volume (LVSV), left ventricular ejection fraction (LVEF), right ventricular stroke volume (RVSV), and right ventricular ejection fraction (RVEF). These calculations adhered to established and standardised formulas, ensuring consistency and accuracy in assessing cardiac function.

Additionally, we recorded the left ventricular mass (LVM) specifically at the end-diastolic phase. In the evaluation of the left atrium (LA), the endocardium was contoured in both the four-chamber and two-chamber views. This dual-contouring approach allowed for the precise determination of the maximum left atrial volume (LAV) just before the opening of the mitral valve, which corresponds to the left ventricular end-systolic phase. The biplane area-length method was employed to assess LA function and dimensions comprehensively. CMR LVFP was derived using LVM and LAV, as previously described, by minor adjustments for the sex of the patient [12].

### 2.4. Statistical Analysis

All clinically acquired data were treated as normally distributed. Continuous variables were then presented using the mean value accompanied by the standard deviation to summarise the data’s central tendency and dispersion. Categorical data, on the other hand, were reported in terms of frequencies and percentages, offering a concise representation of the prevalence of different categories within the dataset. A two-sample independent t-test was used to compare continuous variables. The Chi-squared test was used for categorical data. 

Logistic regression analysis using the Enter method was conducted for a more comprehensive analysis. In this analysis, various CMR assessment parameters and serum levels of NT-proBNP were treated as co-variates, enabling the evaluation of their associations while considering their potential interdependencies. This approach allowed for a thorough examination of how these factors interacted with one another.

Kaplan–Meier analysis and the Cox proportional hazard model were used for multivariate analysis of prognosis. Statistical analysis was performed in SPSS Statistics (IBM, Chicago, IL, USA, version 29) and confirmed in MedCalc (MedCalc Software, Ostend, Belgium, version 22.009). Unless otherwise stated, all statistical tests were two-tailed, and a *p* value of <0.05 was deemed significant.

## 3. Results

### 3.1. Study Population

A total of 380 patients were enrolled in the study. Of the total cohort recruited, 80% (*n* = 305) had NT-proBNP ≥ 125 pg/mL. Patients with raised NT-proBNP levels were older (64 ± 11 years vs. 54 ± 14 years, *p* < 0.0001) and had a higher prevalence of atrial fibrillation compared to those with lower NT-proBNP levels (*p* < 0.01). However, the two groups had no significant disparities in height, weight, or body mass index. Regarding gender distribution, both groups had a similar proportion of males (64%), with no statistically significant difference. The prevalence of diabetes mellitus, hypertension, and hypercholesterolemia was comparable. Regarding pharmacological therapy, substantial distinctions emerged. Patients with NT-proBNP ≥ 125 pg/mL showed a notably higher utilisation of beta-blockers (88%) than the normal NT-proBNP group (61%) (*p* < 0.01). Similarly, aldosterone-converting enzyme inhibitors or aldosterone-receptor blockers (86%), aldosterone-receptor antagonists (36%), diuretics (51%), and oral anti-glycaemic agents (40%) were more frequently administered in the high NT-proBNP group in comparison to the low NT-proBNP group (74%, 16%, 12%, and 12%, respectively) (all *p* < 0.01). All patient demographics and clinical characteristics are summarised in Table 1.

### 3.2. CMR Evaluation

The CMR characteristics of the two groups divided by NT-proBNP levels are shown in Table 2. The group with elevated NT-proBNP levels demonstrated higher LVEDV (221 ± 74 mL vs. 184 ± 46 mL), LVESV (144 ± 70 mL vs. 93 ± 31 mL), and LAV (86 ± 39 mL vs. 65 ± 28 mL), along with a lower LVEF (37 ± 12% vs. 50 ± 8%) compared to the group with normal NT-proBNP levels (all *p* < 0.0001). Additionally, LVM was higher in patients with NT-proBNP ≥ 125 pg/mL (137 ± 42 g vs. 119 ± 34 g), *p* < 0.001). Right ventricular parameters including RVESV (151 ± 54 mL vs. 149 ± 41 mL) and RVEF (47 ± 15% vs. 57 ± 8%) were higher and significantly lower, respectively, in elevated NT-proBNP patients (both *p* < 0.001). However, RVEDV demonstrated no significant difference between the two groups (151 ± 54 mL vs. 149 ± 41 mL, *p* = 0.72).

### 3.3. CMR-Derived LVFP and NT-proBNP

**Figure 1:**
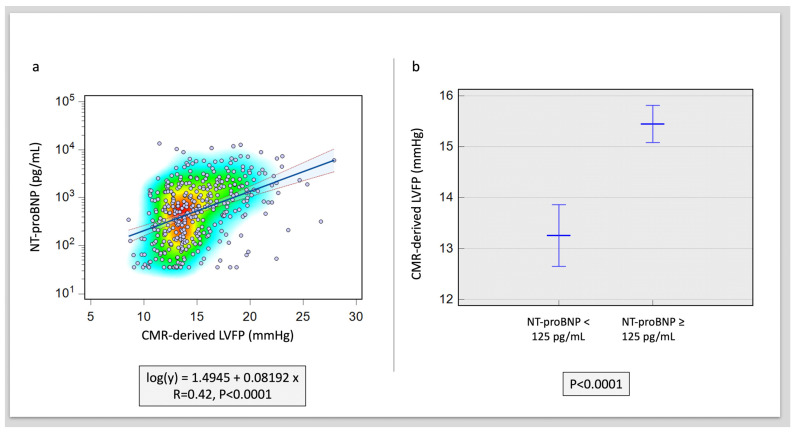
Correlation of NT-proBNP to CMR-derived LVFP. (**a**) Scatter plot with heat map demonstrating the association. (**b**) Difference in CMR-derived LVFP in raised versus normal NT-proBNP levels (*p* < 0.001).

**Figure 2:**
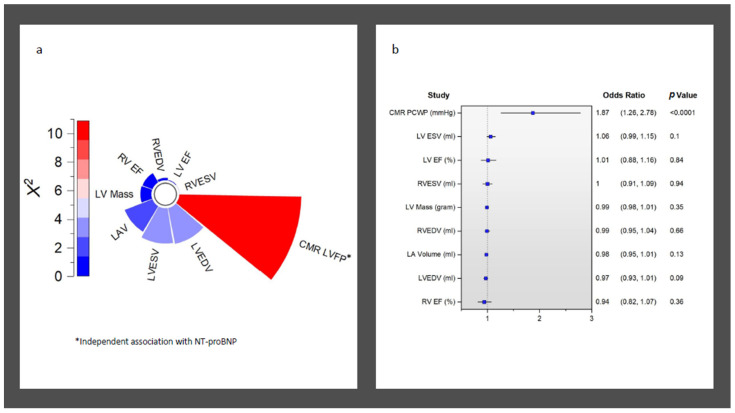
Association of NT-proBNP to all CMR functional and volumetric variables. (**a**) Radial bar chart demonstrating independent association of CMR LVFP to NT-proBNP in logistic regression. (**b**) Odd ratios for all CMR-derived indices associated with raised NT-proBNP.

Overall, CMR LVFP was significantly associated with serum NT-proBNP levels (R = 0.42, *p* < 0.0001) (Figure 1). Furthermore, CMR LVFP was significantly higher in patients with higher NT-proBNP (13.2 ± 2.6 mmHg versus 15.4 ± 3.2 mmHg, *p* < 0.0001) (Table 2). In multivariate analysis, CMR-derived LVFP exhibited an independent association with NT-proBNP while factoring in all other CMR functional and volumetric parameters, including LAV and LVM (χ^2^ = 9.6, Hazard ratio [HR] = 1.87, *p* < 0.001) (Table 3) (Figure 2). Conversely, LVEDV demonstrated a negative but non-significant correlation with an HR of 0.97 (95% CI: 0.93 to 1.01, *p* = 0.09). Similarly, LVESV exhibited a positive yet non-significant correlation (HR = 1.06, 95% CI: 0.99 to 1.15, *p* = 0.10). Other variables, including LV mass, LVEF, LAV, RVEDV, RVESV, and RVEF, did not reveal statistically significant associations (HR = 0.99, 1.01, 0.98, 0.99, 1, and 0.94, respectively) (all *p* > 0.05) (Table 3) (Figure 2).

### 3.4. Survival Analysis for Decompensated Heart Failure

**Figure 3:**
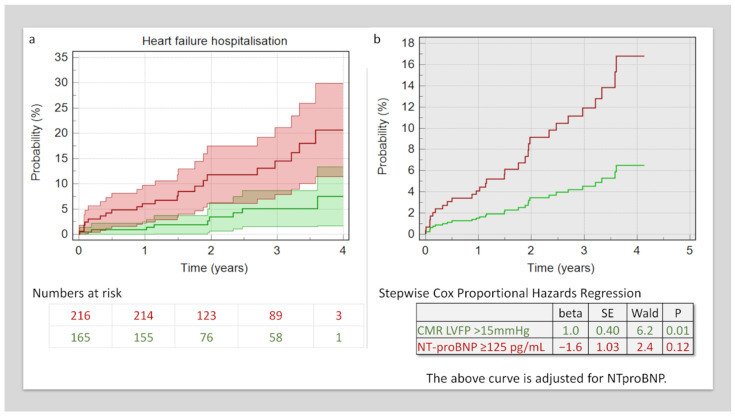
Survival analysis. (**a**) Kaplan–Meier analysis demonstrates patients with raised CMR LVFP had a higher risk of hospitalisation. (**b**) This risk of hospitalisation remained even after factoring in NT-proBNP levels in Cox Proportional Hazard Analysis.

At a mean follow-up period of 2.4 ± 1 years, 30 (8%) of patients were admitted with acute decompensated heart failure. In Kaplan–Meier analysis, patients with higher NT-proBNP had a higher rate of acute decompensation-related hospitalisation (8.3% versus 21%, χ^2^ = 10.3, *p* = 0.001) (Figure 3). In multivariate Cox-proportional-hazard regression factoring in both the NT-proBNP levels and CMR-derived LVFP, only LVFP demonstrated an independent association with HF admission outcomes (beta = 1, standard error = 0.4, HR = 2.7, *p* = 0.01). (Figure 3).

## 4. Discussion

The findings of this study contribute to the existing body of literature on the intricate relationship between NT-proBNP levels, CMR parameters, and their influence on heart failure outcomes. The main observations of this work are that the NT-proBNP levels measured in suspected heart failure patients are associated with CMR LVFP. This association is independent of other CMR parameters, including left ventricular ejection fraction. Moreover, CMR-derived LVFP provides complimentary prognostic information on the risk of HF hospitalisation, which is superior to NT-proBNP. 

Previous studies have explored the individual associations between NT-proBNP levels and various CMR parameters. In patients with hypertrophic cardiomyopathy, NT-proBNP has been found to be a stronger predictor for the diastolic dysfunction assessed by CMR. Chevalier et al. demonstrated an association between NT-proBNP levels and CMR-derived variables (LAV, LVM measured as septal wall thickness and scar volume in grams) in patients with hypertrophic cardiomyopathy [22]. However, they did a multivariate analysis, which included multi-modality parameters. While multivariate analysis that includes multi-modality parameters can provide valuable insights, it comes with inherent challenges related to complexity, data integration, assumptions, dimensionality, generalisability, resource requirements, and clinical implementation. 

The current study introduces a composite marker derived from LAV and LVM as a stronger correlate with NT-proBNP levels, advancing our understanding of heart failure pathophysiology. This approach challenges the traditional reliance on single cardiac parameters and highlights the value of comprehensive assessments for improved risk stratification and tailored interventions. In another study that investigated the association of NT-proBNP with adverse cardiac remodelling in the Multi-Ethnic Study of Atherosclerosis (MESA) population, Rahsepar et al. also demonstrated that the baseline CMR indices were significantly different in increasing the quartiles of NT-proBNP [23]. These findings endorse our observation and, notably, also raise the possibility that CMR LVFP is more strongly associated with adverse LV remodelling than other CMR functional parameters, including LVEF. In a study by Puleo et al. which recruited 1877 patients, NT-proBNP was associated with CMR-assessed LVEDV, wall thickness, and left atrial size [24].

### 4.1. Clinical Implications

The clinical implications of our findings hold the potential to reshape how healthcare professionals assess and manage heart failure patients. The study’s revelation of a strong and independent association between NT-proBNP levels and CMR-derived LVFP highlights the clinical utility of integrating biomarker and imaging data. This association extends beyond traditional parameters like left ventricular ejection fraction, underscoring the importance of considering LVFP as a valuable marker of cardiac health. Importantly, the study’s observation that CMR-derived LVFP surpasses NT-proBNP in predicting the risk of heart failure hospitalisation suggests a more precise and reliable approach to risk stratification. By incorporating CMR LVFP into routine assessments, clinicians may enhance their ability to identify high-risk patients and tailor interventions accordingly, ultimately improving patient outcomes in managing heart failure. Echocardiography remains the first line of the non-invasive assessment of LVFP as it is widely available, cost-effective, and available at the bedside. However, its accuracy can vary, and in cases where further clarification is needed, CMR can be considered instead of invasive assessment. Additionally, while CMR provides comprehensive information about cardiac structure and function, its ability to pinpoint specific causes of heart failure may not consistently surpass a selective strategy based on echocardiography and clinical evaluation. These findings emphasise the need for a comprehensive evaluation of heart failure patients, combining both biomarker and imaging data for more accurate prognostication and personalised care. Future trials need to consider using CMR-derived LVFP rather than echo-derived to measure treatment responses to fully harness the prognostic potential of physiological CMR models.

### 4.2. Limitations

This study, while providing valuable insights, is not without limitations. Firstly, it is a single-centre observational study conducted on individuals recently diagnosed with HF. The timing of the CMR assessments varied among participants, potentially affecting the optimisation of their medical therapy at the time of evaluation. Additionally, since the study’s participants were referred for CMR as part of routine clinical practice, a referral bias may have excluded individuals with greater frailty or those deemed unsuitable for CMR assessment. Furthermore, the absence of invasive catheterisation or concurrent echocardiography data for comparison with CMR-derived left ventricular LVFP limits our ability to fully evaluate the clinical outcomes and the agreement of these different modalities in this population. These limitations underscore the need for a cautious interpretation of the results and highlight potential areas for further investigation.

## 5. Conclusions

A CMR-modelled LVFP is associated with serum NT-proBNP and provides an incremental prognostic value over and above serum NT-proBNP levels.

## Figures and Tables

**Table 1:** Patient demographics and pharmacological treatment stratified by NT-proBNP levels.

	NT-proBNP < 125 pg/mL (*n* = 76)	NT-proBNP ≥ 125 pg/mL (*n* = 305)	*p*-Value
Demographics			
Age, years	54 ± 14	64 ± 11	<0.0001
Height, cms	172 ± 10	170 ± 10	0.12
Weight, kgs	86 ± 20	82 ± 19	0.13
Body Mass Index, kg/m^2^	29 ± 6	29 ± 6	0.36
Male sex, *n* (%)	49 (64)	195 (64)	0.93
Diabetes mellitus, *n* (%)	8 (11)	49 (16)	0.24
Hypertension, *n* (%)	33 (44)	142 (47)	0.69
Hypercholesterolemia, *n* (%)	19 (25)	77 (25)	0.99
Cerebrovascular events, *n* (%)	6 (8)	44 (14)	0.14
Atrial fibrillation, *n* (%)	11 (15)	133 (44)	<0.01
Pharmacological therapy			
Antiplatelets, *n* (%)	15 (20)	54 (18)	0.63
Beta-blockers, *n* (%)	45 (61)	266 (88)	<0.01
Statins, *n* (%)	29 (39)	130 (43)	0.56
Aldosterone-converting enzyme inhibitors or aldosterone-receptor blockers, *n* (%)	55 (74)	262 (86)	<0.01
Sacubitril/valsartan, *n* (%)	3 (4)	11 (4)	0.88
Aldosterone-receptor antagonists, *n* (%)	12 (16)	110 (36)	<0.01
Diuretics, *n* (%)	9 (12)	155 (51)	<0.01
Oral anti-glycaemic agents, *n* (%)	9 (12)	122 (40)	<0.01
Oral anticoagulants, *n* (%)	7 (9)	34 (11)	0.66

**Table 2:** CMR characteristics stratified by NT-proBNP levels.

	NT-proBNP < 125 pg/mL (*n =* 76)	NT-proBNP ≥ 125 pg/mL (*n* = 305)	*p*-Value
CMR characteristics			
Left Heart			
Left ventricular end-diastolic volume, mL	184 ± 46	221 ± 74	<0.0001
Left ventricular end-systolic volume, mL	93 ± 31	144 ± 70	<0.0001
Left ventricular mass, g	119 ± 34	137 ± 42	<0.001
Left ventricular ejection fraction, %	50 ± 8	37 ± 12	<0.0001
Left atrial volume, mL	65 ± 28	86 ± 39	<0.0001
Left ventricular filling pressure, mmHg	13.2 ± 2.6	15.4 ± 3.2	<0.0001
Right heart			
Right ventricular end-diastolic volume, mL	149 ± 41	151 ± 54	0.723
Right ventricular end-systolic volume, mL	65 ± 24	83 ± 43	<0.001
Right ventricular ejection fraction, %	57 ± 8	47 ± 15	<0.0001

**Table 3:** Logistic regression analysis using the enter method demonstrates the independent association between NT-proBNP and CMR variables.

Variable	Coefficient	SE	HR	95% CI	*p*-Value
CMR LVFP	2.02	0.65	1.87	1.26 to 2.78	0.002
LV end-diastolic volume	−2.53	1.49	0.97	0.93 to 1.01	0.09
LV end-systolic volume	4.21	2.53	1.06	0.99 to 1.15	0.10
LV mass	−0.32	0.34	0.99	0.98 to 1.01	0.35
LV ejection fraction	0.18	0.89	1.01	0.88 to 1.12	0.84
LA volume	−0.90	0.59	0.98	0.95 to 1.01	0.13
RV end-diastolic volume	−0.51	1.17	0.99	0.95 to 1.04	0.66
RV end-systolic volume	−0.14	1.93	1.00	0.91 to 1.01	0.94
RV ejection fraction	−0.90	0.99	0.94	0.82 to 1.01	0.36

Abbreviations: CI, confidence interval; CMR LVFP, mean cardiac magnetic resonance imaging measured left ventricular filling pressure; HR, hazard ratio; LA, left atrium. LV, left ventricle; RV, right ventricle; SE, standard error.

## Data Availability

The data underlying this article will be shared upon reasonable request to the corresponding author.
